# Novel insights and new therapeutic potentials for macrophages in pulmonary hypertension

**DOI:** 10.1186/s12931-024-02772-8

**Published:** 2024-03-30

**Authors:** Yifan Zuo, Boyang Li, Minglang Gao, Rui Xiong, Ruyuan He, Ning Li, Qing Geng

**Affiliations:** Department of Thoracic Surgery, Renmin Hospital of Wuhan University, Wuhan, 430060 Hubei China

**Keywords:** Pulmonary hypertension, Macrophage, Hypoxia, Monocrotaline, Animal models

## Abstract

Inflammation and immune processes underlie pulmonary hypertension progression. Two main different activated phenotypes of macrophages, classically activated M1 macrophages and alternatively activated M2 macrophages, are both involved in inflammatory processes related to pulmonary hypertension. Recent advances suggest that macrophages coordinate interactions among different proinflammatory and anti-inflammatory mediators, and other cellular components such as smooth muscle cells and fibroblasts. In this review, we summarize the current literature on the role of macrophages in the pathogenesis of pulmonary hypertension, including the origin of pulmonary macrophages and their response to triggers of pulmonary hypertension. We then discuss the interactions among macrophages, cytokines, and vascular adventitial fibroblasts in pulmonary hypertension, as well as the potential therapeutic benefits of macrophages in this disease. Identifying the critical role of macrophages in pulmonary hypertension will contribute to a comprehensive understanding of this pathophysiological abnormality, and may provide new perspectives for pulmonary hypertension management.

## Introduction

Pulmonary hypertension (PH) is a pathophysiological abnormality characterized by a progressive increase in mean pulmonary arterial pressure (mPAP) over 20 mmHg [1]. PH has been classified into five clinical groups based on clinical and hemodynamic parameters, namely pulmonary arterial hypertension (group 1), PH associated with left heart disease (group 2), PH associated with lung diseases or hypoxemia (group 3), PH caused by chronic thrombotic or embolic disease (group 4), and miscellaneous PH (group 5) [1]. PH is regarded as a major threat to global health issues due to its high prevalence and poor prognosis and is estimated to affect 1% of the global population [2]. The etiologic processes of PH vary from congenital heart diseases, rheumatic heart diseases and infections, which are common in less-developed areas, to respiratory diseases and left heart disorders, which usually affect patients in developed areas [2]. The primary goal of PH treatment is to delay or even reverse this pathophysiological disorder that causes progressive deterioration of the lungs and other target organs, leading to unpredictable and refractory outcomes [1]. Hence, the marked etiological heterogeneity of PH demands precise and targeted medical intervention, which highlights the need for a comprehensive understanding of the pathogenesis of PH.

Pulmonary vascular remodelling is characterized as an important histopathological alteration in PH. Abnormal vascular remodelling of precapillary arterioles, occlusive intimal lesions, and concentric arterial wall thickening have been observed in PH patients. These histopathological alterations are associated with increased pulmonary vascular resistance [3]. A variety of factors, such as genetic context, vascular endothelial cells (ECs), vascular smooth muscle cells (VSMCs), fibroblasts, platelets and inflammatory cells, contribute to the pathogenesis and progression of PH, suggesting the involvement of the whole vessel wall [4]. Recently, alterations in soluble mediator levels as well as perivascular infiltration of immune cells, such as monocytes/macrophages, neutrophils and lymphocytes in established PH have been demonstrated [3, 5–7]. Lekva et al. reported that an elevated level of sCD163, a monocyte/macrophage biomarker, was associated with worse outcomes in PH patients [8]. These results implicate that inflammation plays an important role in PH progression. The infiltration of macrophages occurs mainly in perivascular areas and is involved in the pathogenesis of PH by coordinating the initiation and resolution of pulmonary inflammation [6]. Since macrophages play a critical role in pulmonary vascular remodelling, this review aimed to provide new perspectives for further understanding the pathogenesis and therapeutic strategies for this catastrophic condition. Group 2, Group 3, and Group 4 PH are often included and discussed in their primary conditions, such as left heart dysfunction or chronic lung diseases. Therefore, we focused mainly on Group 1 (pulmonary arterial hypertension) in this review.

### Origin of macrophages in the lung during PH

There are two types of tissue-resident macrophages in the lung, alveolar macrophages (AMs) and interstitial macrophages (IMs). AMs originate from embryonic liver mononuclear cells, reside on alveoli and airways and maintain immune homeostasis [9]. IMs originate from circulating monocytes and participate in adaptive immune responses via interactions with interstitial T lymphocytes [10]. Mononuclear phagocytes are recruited to the lung during infection and then shift toward different subtypes [11]. It is currently accepted that macrophages can be classified into two main subgroups: the classically activated type (M1), which promotes the inflammatory response via the synthesis and release of proinflammatory cytokines; or the alternatively activated type (M2), which promotes inflammation resolution and tissue repair [12]. Montani et al. reported the involvement of bone marrow-derived CD117 + cells in human idiopathic PH [13]. Circulating monocytes are recruited to the lungs when chemokine levels increase, where they replace resident interstitial macrophages and subsequently participate in vascular remodelling [14]. Therefore, monocytes are recruited and then react to microenvironmental changes in the lungs during external or internal stimuli, and function as key cellular mediators to coordinate inflammatory reactions, differentiation and polarization into different active subtypes [15].

### Macrophages infiltrate vessels during PH, contributing to vascular remodelling

The remodelling of pulmonary arterial vessels is essential for PH progression, and leads to a progressive increase in mPAP [3]. Arterial remodelling requires the accumulation of resident pulmonary vessel cells and inflammatory cells, which suggests the involvement of all three layers. Specific pathological features, such as fibrosis alterations, can be detected via histopathological assessment at the early stage of PH progression [3]. Both external and internal stimuli, such as genetic mutations, hypoxia, cold exposure, air pollution, and respiratory infection, can initiate immune responses and therefore lead to the proliferation of vascular cells, autoantibody formation, and dysregulated immunity [16–20]. Histopathological evidence has suggested that perivascular infiltration of inflammatory cells is common in PH and precedes structural remodelling in vessels [5, 6, 17, 21, 22]. In idiopathic PH, the level of immune infiltration is higher for CD8 + T cells, resting memory CD4 + T cells, γδ T cells, M1/M2 macrophages and resting mast cells; and lower for monocytes, neutrophils and naïve CD4 + T cells [23–25]. These results suggest that immune imbalance contributes to remodelling in PH.

Hypoxia-induced (sugen, a vascular endothelial growth factor antagonist, is used simultaneously in many studies) and monocrotaline (MCT)-induced experimental PH models, which are similar to group one pulmonary arterial hypertension, have been widely adopted [26, 27]. Compared with human PH samples, experimental PH models provide richer information about macrophage infiltration. Monocyte numbers increase in both the bone marrow and the blood when hypoxic exposure is prolonged [28]. After one day of exposure to hypoxia, the IL-1β and oncostatin M regulation-related subtypes of macrophages are the most abundant groups in the lungs of mice, which are often associated with proinflammatory effects. Later, new subtypes that are overrepresented with mitochondrial dysfunction, oxidative phosphorylation and the EIF2 signalling pathway emerge after seven days of hypoxic exposure; these subtypes are involved in anti-inflammatory effects and tissue repair [29]. As Pugliese et al. reported previously, during the early period of hypoxic exposure, macrophages accumulate around pulmonary vessels, exhibit a hypoxic response and release proinflammatory cytokines; subsequently, the perivascular accumulation of macrophages decreases and demonstrates the tissue repair and anti-inflammatory programming states [30]. Inhibiting the early accumulation of monocytes and macrophages has been reported to effectively ameliorate the right ventricular burden and Fulton index in hypoxic mouse models [31]. The genetic/pharmacological intervention of several key signalling pathways in macrophages can protect against experimental PH progression [32–41]. These potential targets function via different mechanisms, such as the alleviation of macrophage-related pulmonary inflammation and the inhibition of macrophage activation and polarization. Additionally, the depletion of AMs reportedly attenuates hypoxia-induced PH in rats [42]. These genetic or pharmacological targets are summarized in Table 1.

**Table 1:** Genetic or pharmacological targets associated with macrophages in the PH model

Author	Year	Main result	Model	Species	Intervention	Reference
Yaku et al.	2022	Regnase-1 regulates IL-6 and PDGF in alveolar macrophages.	Hypoxia	Mouse	Genetic knockout	[41]
Yu et al.	2022	Selective BTK inhibitor BGB-3111 regulates macrophage recruitment and polarization.	MCT	Rat	BGB-3111	[36]
Rong et al.	2022	Caspase-8 deletion or pharmacologically blocking affects the proinflammatory factors secreting in M1 macrophages.	SU5416/Hypoxia, MCT	Mouse, rat	Genetic knockout and inhibitor (Z-IETD-FMK)	[38]
Kojima et al.	2019	HIF-1α deletion in myeloid suppresses macrophage infiltration.	Hypoxia	Mouse	Genetic knockout	[33]
Hu et al.	2019	Hif-2 inhibitor PT2567 attenuated monocyte recruitment.	Hypoxia	Rat	PT2567	[35]
Xi et al.	2019	SGK1 knockout inhibits proinflammatory cytokines expression and inflammatory infiltration of macrophage.	Hypoxia	Mouse	Genetic knockout	[39]
Amsellem et al.	2017	Inactivation of CX3CR1 modulates monocyte recruitment and macrophage phenotype.	Hypoxia	Mouse	Genetic knockout and CX3CR1 antagonist F1	[40]
Barman et al.	2014	Nox4 colocalizes with monocyte markers. Nox4 inhibitor VCC202273 attenuates PH progression.	SU5416/Hypoxia, MCT	Mouse, rat	VCC202273	[37]
Tian et al.	2013	Blocking macrophage Leukotriene B4 abrogates endothelial injury.	SU5416/Hypoxia, MCT	Rat	LTA4H inhibitor Bestatin	[32]

Abbreviations: IL-6, Interleukin-6; BTK, Bruton’s tyrosine kinase; MCT, monocrotaline; HIF-1α, hypoxia-inducible factor-1α; SGK1, serum glucocorticoid-regulated kinase-1; LTA4H, leukotriene A4 hydrolase

Recently, unexpected macrophage infiltration has been detected in the right ventricle in MCT- or hypoxia-induced rat models, leading to inflammation in the right heart [43, 44]. Therefore, the inflammatory response might not only affect the lungs and pulmonary vessels, the heart can also suffer from similar pathophysiological alterations. The similar macrophage infiltration in the heart is important too, because right heart dysfunction and failure are major risk factors for poor prognosis in PH patients. Interestingly, the depletion of macrophages reportedly induces experimental PH in male mice but not in female mice [45], suggesting a potential association between sex and immune cells in PH. This is quite interesting, since PH is more likely to be a female predominant disease with a female-to-male ratio of approximately 4:1, while PH tends to be more severe in males [46]. Oestrogen is believed to mediate the protective effect of female sex in PH via oestrogen receptor-β, based on evidence from MCT-induced rat PH models treated with oestrogen [47]. The genetic variation in oestrogen metabolites and the canonical/noncanonical TGF-β signalling pathways regulated by sex hormones might explain the female predominance in PH [48, 49]. For example, oestrogen inhibition induced by aromatase inhibitors can reduce the development of PH in bmpr2-mutant mice [49]. Oestrogen can regulate the expression of TGF-β receptors and signalling modulators, such as endoglin and TGF-β3, at the transcriptional and protein levels [48]. Other sex hormones, including progestogen, androgen and anti-Müllerian hormone might also contribute to the sex-related differences in PH. Therefore, oestrogen metabolism and oestrogen receptors are regarded as promising therapeutic targets in PH [50]. Additionally, elevated aromatase expression is elevated in the special phenotype of female pulmonary SMCs, suggesting a higher level of estradiol synthesis and thus contributing to female PH susceptibility [51]. Regulatory T-cell function has been demonstrated to be a protective factor in female rodent models of PH, that Treg deficiency results in more severe PH in females than in males [52]. The Y chromosome was reported to be protective against the development of hypoxia-induced PH [53]. However, oestradiol supplementation can also limit several maladaptive processes in the right ventricles associated with PH, such as pro-apoptotic signalling, oxidative stress and activation of pro-inflammatory cytokines, which might prevent right ventricle remodelling [49]. Until now, no sex difference was found in macrophage polarization [45], and there is no direct evidence that macrophages act differently in different sexes.

### Macrophages react to PH triggers and polarize to different subgroups

**Fig. 1:**
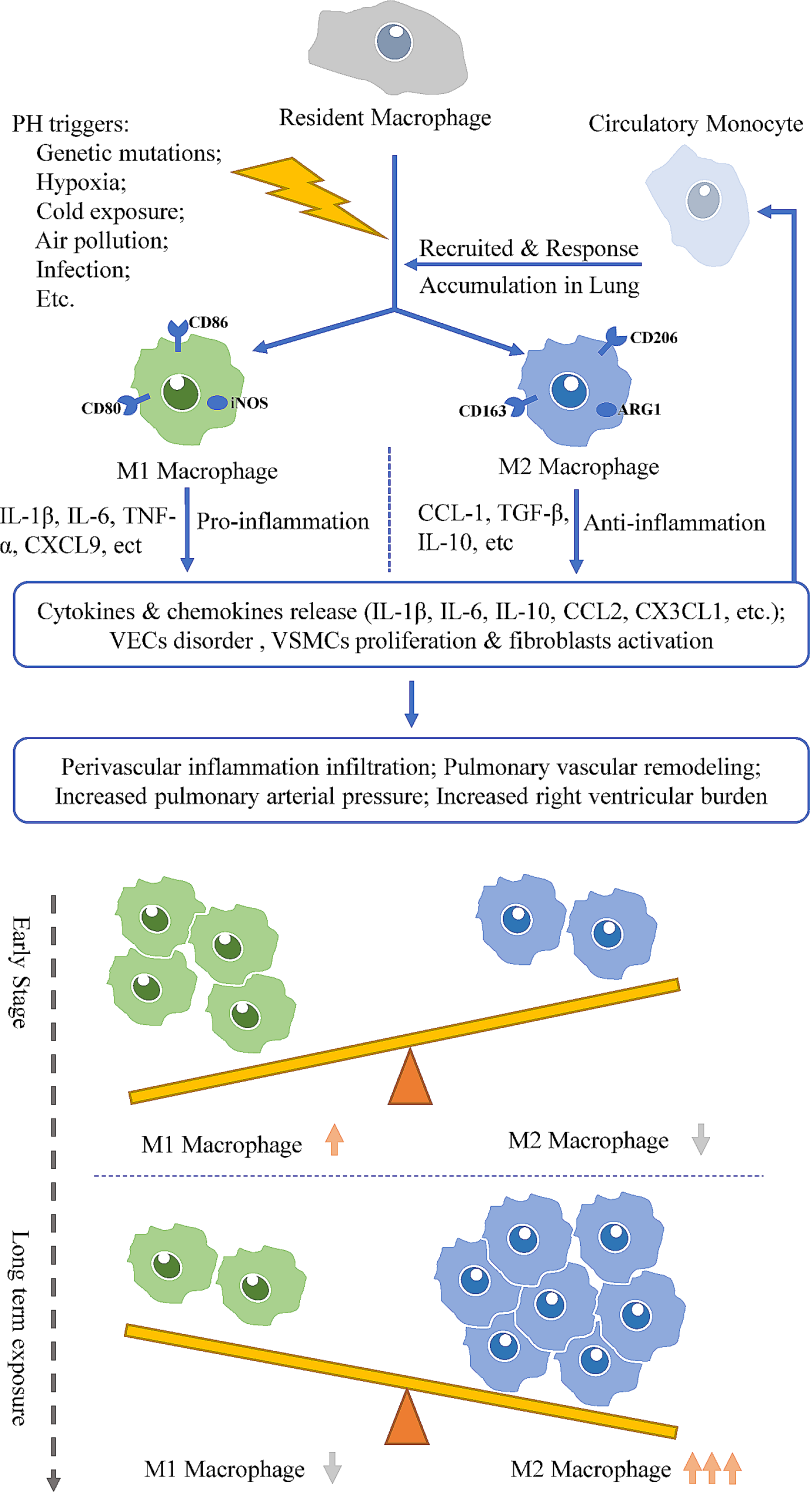
PH triggers induce macrophage activation and polarization. During the early stage, recruited and resident macrophages polarize to the M1 type in response to external stimuli. Then M2 type dominates the main polarization type of macrophages in the lung for tissue repair, as stimuli are constantly present. Macrophage activation and polarization are complex and consecutive processes, that cannot be simply regarded as separate and contradictory processes involved in PH progression

As discussed previously, macrophages infiltrate the perivascular area during PH. Monocyte-derived macrophages are recruited to the lung and then differentiate in response to microenvironmental changes during pulmonary damage; these cells are involved in lung injury and repair [15, 54]. The time- and compartment-specific activation of lung macrophages was reported by Pugliese et al. [30]. During the early stage of hypoxic exposure, macrophages accumulate vastly around pulmonary arterioles instead of the alveolar space, with upregulation of mTORC1 signalling, glycolysis, and oxidative phosphorylation in both AMs and IMs. The expression of IL-1, IL-5, CCL-5, and EGFR is also increased in both AMs and IMs during the early stage of hypoxic exposure. When hypoxia is prolonged, the perivascular accumulation of macrophages decreases and presents different compartment-specific activation, that perivascular IMs tend to signal against inflammation and promote tissue repair/remodelling while AMs still exhibit high proinflammatory-related signalling [30]. A typical change in macrophage subgroups in the experimental PH model is that M1-polarized macrophages predominate in the early phase after stimulation; then, M2-polarized macrophages accumulate in the lung with an increase in the M2/M1 ratio to almost ten after four weeks of induction [28, 55, 56]. M1 macrophages are regarded as proinflammatory components, while M2 macrophages are often associated with anti-inflammatory and reparative effects [45]. However, interruption of the M1 subgroup can also effectively protect against PH [38]. Additionally, a rebalanced M2/M1 ratio could be observed in alleviated PH models [57]. Changes in macrophage activation and the M1/M2 ratio are presented in Fig. 1.

The activation of macrophages in the hypoxia-induced experimental PH model has been reported to be related to alterations in several signalling pathways and cytokines, such as hypoxia-inducible factor 1 (HIF-1), IL-6, NF-κB, HIMF and IL-6 [58]. During the early phase of hypoxia, the increased expression of Ythdf2 in AMs promotes the degradation of m6A-modified Hmox1 mRNA and the activation of macrophages; consequently, levels of anti-inflammatory mediators (such as IL-10) are evaluated and promote the proliferation of VSMCs, leading to PH progression [59]. The deletion of Ythdf2 in bone marrow-derived macrophages (BMDMs) leads to the promotion of the M1 subtype by enhancing MAPK and NF-κB signalling and the inhibition of the M2 subtype by upregulating p53 expression [60]. This result provides another potential explanation for why the absence of Ythdf2 in macrophages protects PH. IMs express thrombospondin-1 after hypoxic exposure, thus promoting hypoxic PH progression via TGF-β activation [61].

Human RELM-β, which is homologous to the rodent RELM-α (also known as HIMF or FIZZ1) and is regarded as a marker of the M2 subtype, has been reported to be upregulated in the proliferative stage in hypoxia-induced PH [62]. HIMF-positive signals are mainly colocalized with perivascular macrophages [63]. When exposed to chronic hypoxia, RELM-α emerges in macrophages and subsequently promotes the activation and release of HMGB1, while the DAMP receptor RAGE is required for HMGB1 function and maintenance of inflammation. Hence, an autocrine positive feedback loop is established in macrophages, resulting in continuous vascular inflammation and proliferation of VSMCs in PH [64]. The hypoxia-induced increase in RELM-β could also inhibit membrane KCNK3 expression via PLC activity and endocytosis [65], which have been reported to contribute to local inflammatory response, vascular remodelling and proliferation [66]. The IL-4 signalling pathway can synergistically enhance the HIMF-induced expression of vascular endothelial growth factor (VEGF) and monocyte chemoattractant protein-1 (MCP-1), which is also associated with angiogenesis in pulmonary microvessels [67, 68].

Hypoxia-inducible factor (HIF) is a major regulator of oxygen homeostasis that initiates a series of hypoxic responses, including changes in vascular tone and proliferation, cellular metabolism, autophagy and cellular survival [34]. HIMF induces the recruitment of macrophages to the angiogenesis area in the HIF-1-dependent manner and the production of IL-1 by perivascular macrophages and VSMCs in the HIF-1α-dependent manner [62]. Deletion of HIF-1α in BMDMs reduces right heart remodelling and pulmonary macrophage infiltration in hypoxia-induced PH models, suggesting the involvement of HIF-1 in PH progression [33]. It has also been reported that decreased perivascular macrophage infiltration in hypoxia-induced PH models was associated with the alleviation of pulmonary vascular muscularization [33]. Leptin, a HIF-dependent peptide hormone, is reportedly upregulated in pulmonary vessels, leading to abnormal monocyte/macrophage activation, perivascular macrophage accumulation, and IL-6 overexpression [69, 70]. Hence, interfering with macrophage migration seems to be a promising target for PH treatment. A previous report indicated that HIF-1α is expressed at higher levels in M1 macrophages, and HIF-2α is expressed at higher levels in M2 macrophages [71]. The suppression of HIF-2α can alleviate hypoxia-induced PH [35]. Active arginase can reduce the supply of L-arginine, which is needed for NO synthase [72], thus decreasing NO production in macrophages. Arginase 1 expression in macrophages is induced by HIF-2α [73]. The protective effect of HIF-2α suppression in PH might be related to M2 macrophage dysfunction, because NO contributes to vascular resistance regulation and M2 polarization contributes to vascular remodelling.

In the MCT-induced rat model, Bruton’s tyrosine kinase (BTK), a nonreceptor tyrosine kinase of the Tec family that is linked to B-cell proliferation and survival, is upregulated in the lung and colocalized with CD68 + macrophages [36]. BTK inhibitors can prevent M1 polarization and PH progression in experimental PH models [36]. In addition, deficiency of legumain (also known as asparaginyl endopeptidase) in macrophages has been reported to attenuate hypoxia-induced PH in mouse models [74].

### Activation and metabolic reprogramming in macrophages

Leukotriene B4 (LTB4) is released by activated macrophages and can induce pulmonary arterial endothelial apoptosis and extensive vascular injury [32]. LTB4 can also promote the proliferation, migration, and differentiation of fibroblasts through both the activation of p38 MAPK signalling and the upregulation of Nox4 [75]. Nox4 and reactive oxygen species (ROS) are detected in the adventitia and overlap with fibroblast markers (fibroblast activating proteins) and a monocyte marker (CD11b) [37]. Nox4-derived ROS activate the transient receptor potential melastatin 2 and therefore enhance the proliferation and migration of VSMCs in pulmonary arteries [76]. M1 macrophage infiltration in the right ventricle with enhanced NOD-like receptor thermal protein domain associated protein 3 (NLRP3) expression and activation results in right heart dysfunction [43]. Stimulators of interferon genes in macrophages induce PH progression via the activation of NLRP3 signalling transduction [77]. Moreover, prostaglandin D2 released by macrophages inhibits smooth muscle cell proliferation and induces vasodilation [78]. Under particular conditions, extracellular vesicles that are released by macrophages in certain infections carry high levels of TGF-β1, which is associated with increased pulmonary arterial systolic pressure [79].

**Fig. 2:**
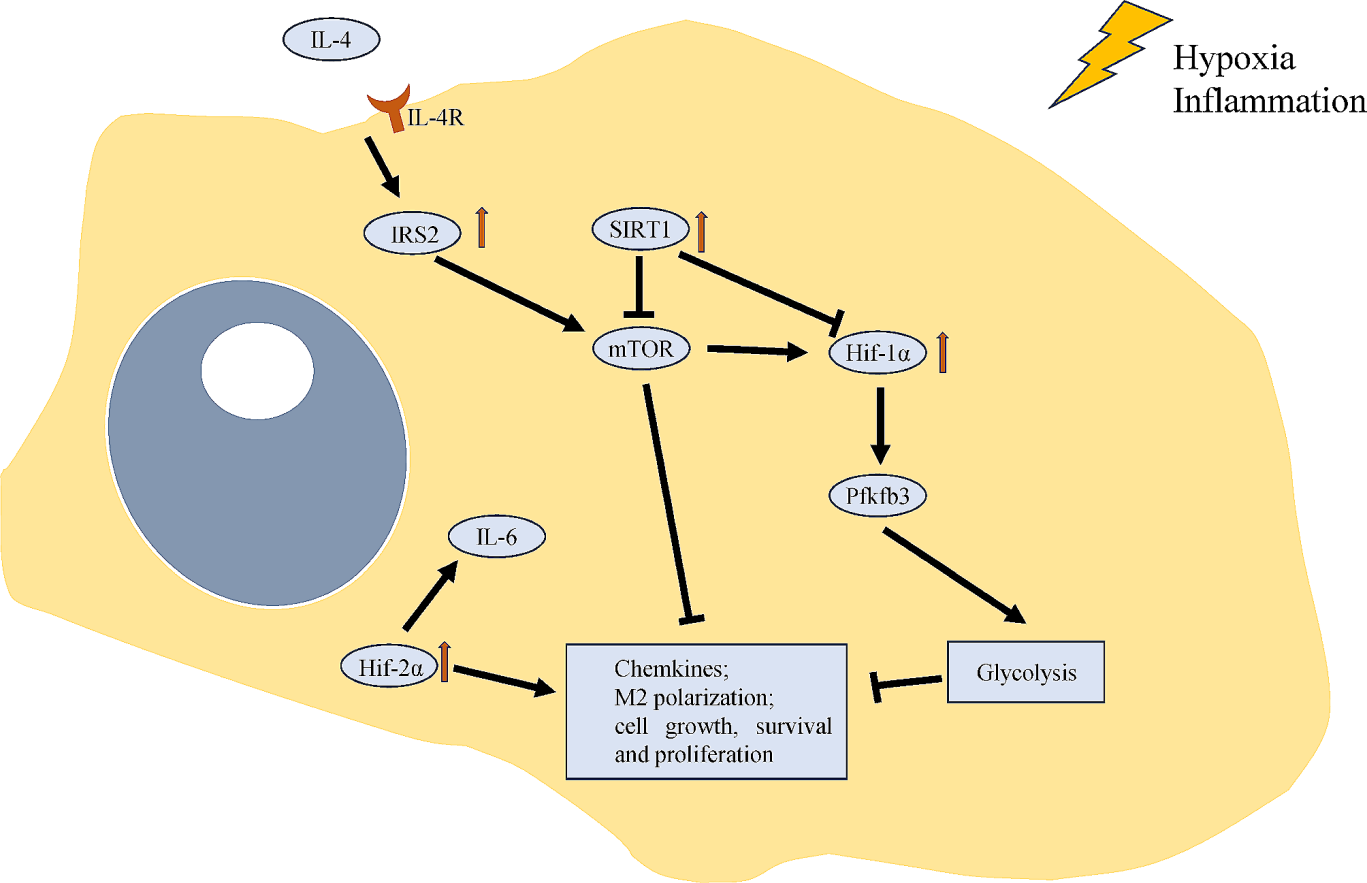
Metabolic reprogramming in macrophages exposed to hypoxia and inflammation. Both HIF-1α and HIF-2α were increased by hypoxia, leading to different responses in macrophages. Glycolysis usually characterizes M1 macrophages and suppresses M2 polarization, while HIF-2α promotes cell survival and the expression of specific cytokines, such as IL-6. Circulating cytokines such as IL-4, and SIRT1 can also affect metabolic reprogramming in macrophages by activating or inhibiting mTOR signalling

When exposed to inflammatory stimuli or chronic hypoxia, macrophages are activated and then undergo aerobic glycolysis [80], which in turn governs macrophage function [81]. Metabolic reprogramming occurs in various cells in PH, including VECs, VSMCs, fibroblasts and immune cells, and plays a synergistic role with other PH hallmarks such as proliferation, apoptosis resistance and inflammation [82]. In macrophages, this metabolic shift is usually reversible and might involve the synthesis and secretion of immune mediators [83]. The metabolic shifts are mainly induced by HIF families and prolong survival under stress [84]. For example, HIF-1α is induced in M1-macrophages, while glycolytic and pentose phosphate pathways are also enhanced; in M2-macrophages, HIF-2α is elevated, while fatty acid oxidation level and mitochondrial respiratory chain activity are also increased [85]. These metabolic shifts are important for vascular diseases [86]. Metabolite alterations and the mitochondrial electron transport chain distribution characterize reprogrammed macrophages [87]. Chronic hypoxia might lead to a prolonged glycolytic shift even when the stimulus has been removed [84]. Due to the angiogenesis and chronically inflammatory nature of PH, several studies have attempted to alleviate PH by interfering with metabolic reprogramming. G6PD activity inhibitors decreased the accumulation of macrophages in hypoxic PH mice [28]. The specific deficiency of Pfkfb3 in myeloid cells, that Pfkfb3 is a critical enzyme of macrophage glycolysis, protects mice from PH and decreases the levels of growth factors and proinflammatory cytokines in experimental PH models [88]. When exposed to IL-4 and hypoxia, the level of insulin receptor substrate 2, a critical regulator of cellular energy homeostasis, is decreased in macrophages and subsequently contributes to chronic inflammation and vascular dysfunction [89]. SIRT1-mTOR/HIF-1α signalling can promote M2-type differentiation by blocking glycolysis and can reduce the recruitment of inflammatory cells [90, 91]. Macrophages reprogrammed by HIF-2α can produce several specific cytokines, such as IL-6, thus protecting organs from injury [92]. The activation of mTOR and HIF-1α can be induced by many factors, such as infection and the circadian clock [93, 94]. However, proinflammatory cytokine production within macrophages might not be glycolytic reprogramming-dependent [95]. In Table 2; Fig. 2, we summarize the published studies on experimental PH that focused on macrophages. Although metabolic reprogramming is an important characteristic of activated macrophages, it can occur in many other cellular components, such as fibroblasts and vascular endothelial cells, during PH progression.

**Table 2:** Recent studies focused on macrophages in PH models

Studies	Year	Models	Main results	Role of macrophage in PH
Wu et al. [77]	2023	Rat model (hypoxia)	STING was mainly colocalized with CD68^+^ macrophages. STING inhibition prevented the overactivation of NLRP3 signalling and macrophage activation in rat models.	Macrophage activation in PH.
Chi et al. [96]	2022	Rat model (MCT, hypoxia)	Elevated MMP-1 and MMP-10 in M1 macrophages. MMP-10 promoted proliferative and pro-migratory phenotypes of VSMCs.	Macrophage regulates VSMC behavior in PH.
Jeong et al. [97]	2022	Mouse model (hypoxia)	Inhibition of RUNX1 dampens macrophage recruitment and activation.	Macrophage activation in PH.
Yu et al. [36]	2022	Rat model (MCT)	BTK was upregulated and mainly colocalized with macrophages. BTK inhibition suppressed recruitment of M1 polarized macrophage and vascular remodelling in MCT-induced PH.	Macrophage activation and polarization in PH.
Gu et al. [98]	2022	Mouse model (hypoxia, schistosomiasis)	Macrophage subpopulation infiltration increased in the right ventricle after acute hypoxia: CD11c^low^MHCII^low^ and CD11c^high^MHCII^high^.	Macrophage subgroups in PH.
Wang et al. [88]	2021	Mouse and rat model (hypoxia)	Pfkfb3 regulated the expression of proinflammatory cytokines and growth factors. PH was ameliorated when Pfkfb3 was suppressed.	Macrophage activation in PH.
Nakahara et al. [89]	2021	Rat model (hypoxia)	IRS2 was downregulated by IL-4 and hypoxia stimulation in macrophages. IRS2 negatively regulated Akt and ERK pathways in macrophages.	Macrophage activation and polarization in PH.
Ntokou et al. [99]	2021	Mouse model (hypoxia)	PDGFb upregulated in macrophages. PDGFb-induced pathological smooth muscle cell expansion.	Macrophage regulates VSMC behavior in PH.
Kumar et al. [61]	2020	Mouse model (hypoxia)	Interstitial macrophages expressed thrombospondin-1 after hypoxia.	Macrophage regulates vasoconstriction in PH.
Batool et al. [100]	2020	Mouse and rat model (hypoxia)	Expression of Stamp2 decreased in macrophages after hypoxic exposure. Stamp2 deficiency led to aggravated pulmonary inflammation and worsening of hypoxia-induced PH.	Macrophage activation in PH. Macrophage regulates VSMC behavior in PH.
Park et al. [56]	2020	Rat model (MCT)	Uptake of macrophage infiltration tracker ^68^Ga-NOTA-MSA in the lung was observed in both rat PH models and PH patients.	Macrophage infiltration in PH.
Xi et al. [39]	2019	Mouse model (hypoxia)	Increased SGK1 expression in macrophages. SGK1 deficiency inhibited macrophage activation and inflammatory response.	Macrophage activation in PH.
West et al. [101]	2019	Mouse model (hypoxia, BMPR2 knockdown)	BMPR2 suppression in macrophages contributed to pulmonary vascular remodelling.	Macrophage activation in PH.
Yin et al. [102]	2017	Rat model (MCT)	Activation of the P2 × 7R in macrophages. P2 × 7R inhibition suppressed cytokine levels and ameliorated vascular remodelling in PH.	Macrophage activation in PH.
Saito et al. [103]	2017	Rat model (Single Su5416)	A high level of HERV-K expression was observed in perivascular CD68^+^ cells in PH patients. HERV-K dUTPase was induced by inflammatory stimuli and caused pulmonary hypertension in rat models.	Macrophage activation induced by endogenous retrovirus in PH.

Abbreviations: STING, Stimulator of interferon genes; MMP, matrix metalloproteinase; RUNX1, Runt-related transcription factor 1; BTK, Bruton’s tyrosine kinase; Pfkfb3, 6-phosphofructo-2-kinase/fructose-2,6-bisphosphatase 3; IRS2, insulin receptor substrate 2; SGK1, serum glucocorticoid-regulated kinase-1; BMPR2, bone morphogenetic protein receptor type 2; P2 × 7R, P2 × 7 purinergic receptor; HERV-K, human endogenous retrovirus K

### Interaction of macrophages with soluble mediators

The expression of many immune mediators is altered in PH, including cytokines (such as IL-1β, IL-6, IL-10, and IL-18), chemokines and their receptors (such as CCL2 -CCR2, CCL5-CCR5, CXCL12, CX3CL1, LTB4), HIMF, and complement [7, 104]. Exosomes from mesenchymal stem cells can also regulate pulmonary inflammation via modulation of macrophage function [15, 57]. These soluble components are involved in both the inflammatory response and tissue repair, as well as interactions between different cells. Several proinflammatory or anti-inflammatory biomarkers have been applied to predict the outcome of PH clinically or experimentally [105]. In Table 3, we summarize the origins of these mediators and the cell types affected. Most mediators are produced by both macrophages and other cellular components (such as fibroblasts or smooth muscle cells) during PH progression. The levels of several chemokines, such as CCL2 and CCL5, are reportedly increased in proinflammatory fibroblasts and can induce macrophage transmigration. Together, these mediators, which have various origins, regulate PH progression.

**Table 3:** The origins and targets of the main soluble mediators in pulmonary hypertension

	Produced by	Affects
IL-1	Fibroblast, macrophage, neutrophil, T cell, VSMCs	VSMCs, macrophage
IL-6	Fibroblast, VSMCs, Th1 cell, macrophage	Macrophage, Th1 cell, Th2 cell
IL-8	VSMC, macrophage, VECs	Neutrophil
IL-10	Th2 cell, B-cell	Macrophage, Th1 cell, VSMCs
MIF	Fibroblast, macrophage, VECs	VSMCs, VECs
CCL2	Fibroblast, VECs, VSMCs	Macrophage
CCL5	Fibroblast, VECs	Macrophage
CX3CL1	Macrophage, VECs	Macrophage, VSMCs
CXCL12	Fibroblast, VECs	Macrophage

Abbreviations: IL, Interleukin; VSMCs, vascular smooth muscle cells; VECs, vascular endothelial cells; MIF, migration inhibitory factor

### IL-6

IL-6 has diverse cellular origins, such as fibroblasts, macrophages, pulmonary arterial smooth muscle cells, and Th1 lymphocytes [6, 7, 16]. In the very early stage of hypoxia or MCT exposure, increased levels of both IL-6 protein and mRNA can be detected in experimental PH model lungs [106, 107]. IL-6 plays a complex role in inflammation. IL-6 has both a proinflammatory effect by stimulating IL-4 production in Th2 cells and an anti-inflammatory effect by inhibiting IFN-γ production in Th1 cells [16]. Fibroblasts subjected to hypoxic conditions exhibit resistance to apoptosis in response to environmental changes [82]. The IL-6/STAT-3 signalling contributes to macrophage activation in hypoxia-induced PH models [108]. The IL-6/IL-21 axis is involved in M2 polarization, and IL-6 blockade inhibits M2 polarization in the hypoxia-exposed mouse model of PH [108]. Macrophage-derived IL-6 has been reported to promote pulmonary vascular remodelling [34]. The pharmacologic blockade or mRNA degradation of IL-6 in AMs can decrease right ventricular systolic pressure and alleviate PH progression in hypoxic mouse models [41].

### IL-10

CD4 + Th2 cells, B lymphocytes and M2 polarized macrophages produce IL-10 [109]. IL-10 inhibits the proliferation of smooth muscle cells and the synthesis of proinflammatory cytokines in macrophages and Th1 lymphocytes [16]. Upregulation of IL-10 expression in PH rat models alleviated inflammatory infiltration and VMSC proliferation, as well as mPAP and right ventricular hypertrophy [110]. The protective effect of increased IL-10 levels in PH models was reported with alterations in other PH-related cytokines [31]. Hence, quantifying the protective effect of IL-10 in PH seems unlikely. Nevertheless, IL-10 is still a promising target for PH intervention.

### IL-1

IL-1 is produced by diverse cell types, including monocytes, fibroblasts, T lymphocytes, neutrophils, and even pulmonary arterial smooth muscle cells [6, 7, 29, 30]. Upregulation of IL-1 occurs in the very early period of PH initiation and progression in response to hypoxia in macrophages [29]. Through the IL-1β/IL-1R1/MyD88 pathway, macrophages induce the proliferation of VSMCs in pulmonary arteries, suggesting that IL-1β is involved in pulmonary vascular remodelling in experimental PH [111].

### IL-8 and migration inhibitory factor

IL-8 is expressed by macrophages or injured vascular cells. In the MCT-induced PH models, neutrophil infiltration and proinflammatory mediator expression are reduced by the upregulation of IL-8 in VECs [112]. The migration inhibitory factor (MIF) produced by T-cell lymphocytes is increased and activated in idiopathic PH patients, while an antagonist of MIF can partially reverse the development of experimental PH [113]. MIF functions as a main proinflammatory cytokine and VSMC proliferation promoter, and MIF expression and secretion levels are elevated in fibroblasts, monocytes, and endothelial cells after infection or hypoxia [114].

### Chemokine

CCL2-CCR2 and CCL5-CCR5 are essential for the initiation and amplification of VSMCs in PH pathogenesis [115]. Increased expression of CCL2 and its ligand CCR2 was observed in the lungs of hypoxic PH models [40]. Proinflammatory fibroblasts in the vascular adventitia under hypoxic conditions express high levels of CCL2, CCL5, and CXCL12 [6, 116]. CCR2 is essential for the recruitment and development of M1-polarized macrophages, while increased CCL5 induces transmigration, adhesion, and activation [6, 11, 116]. Bordenave et al. reported a marked alleviation in distributed pulmonary hemodynamics and structural disorders in both the lung and heart when chemokine CXCL12 was neutralized in rat PH models [117]. Furthermore, CXCL12 neutraligand administration (chalcone 4, LIT-927, and AMD3100) decreased macrophage infiltration in the lungs of PH rat models [117]. In addition, the expression of CX3CR1 and its ligand CX3CL1 has been reported to increase in the lungs of hypoxia-induced PH models [40]. The inhibition of the CX3CL1-CX3CR1 signalling pathway effectively attenuates pulmonary inflammation and arterial remodelling, thus leading to a certain degree of improvement in hemodynamics [14]. Genetic deletion or pharmacological inhibition of CX3CR1 prevents hypoxia-induced PH by regulating monocyte recruitment, macrophage polarization and VSMC proliferation, which are associated with a changed balance between the M1 and M2 phenotypes [40]. These findings highlight the close interaction between CX3CL1 and macrophages, suggesting that the CX3CL1-CX3CR1 signalling pathway is a potential therapeutic target for PH.

Although these mediators are involved in the promotion or prevention of PH pathogenesis and progression, it is unwise to simply define them as “harmful” or “beneficial”. There is a complex and ingenious collaboration between mediators and cellular components. Cytokines and other inflammatory factors are synthesized and released by both macrophages and other cellular components, and subsequently activate or repress specific downstream signalling pathways, which in turn initiate pro- and/or anti-inflammatory responses and induce the activation or repression of other inflammation-related cells, such as immune cells, fibroblasts, ECs, and even smooth muscle cells. For example, PH patients with moderate cytokine levels have the best prognosis, while groups with the strongest or lowest immune signals have more severe clinical symptoms and worse outcomes [118], despite disordered circular inflammation factors being quite common in PH patients.

### Interactions of macrophages with other cellular components in PH

Cellular abnormalities, including pulmonary vascular endothelium dysfunction, VSMC and adventitial fibroblast accumulation in arteries, and innate/adaptive immune system dysregulation, are critical promotors in PH [17]. Pulmonary ECs are transformed into proinflammatory phenotypes, then produce and release multiple cytokines and chemokines, leading to changes in endothelial communication between other resident vascular cells and circulating cells. Endothelial dysfunction and environmental stress induce metabolic alterations and proliferation in pulmonary VSMCs and fibroblasts. Moreover, perivascular inflammatory infiltration and circular cytokine alterations can be detected in the early stage of PH, suggesting that the immune system responds to microenvironmental changes and external stimuli even before substantial vascular remodelling occurs [17, 119]. These changes interact with other PH promotors.

Histological evidence has indicated concentric or eccentric intima-media thickening in muscularized precapillary arteries in individuals with established PH [3], which highlights the involvement of ECs and smooth muscle cells. ECs are essential for the initiation of PH. HIF-2α is activated in ECs under hypoxia, which is required for prominent proinflammatory genes Sdf1 (CXCL12), CXCR4, ICAM1 and VCAM1; then monocytes/macrophages and other circulation-/bone marrow-derived cells are recruited to lung at the early stage of PH development [35, 120]. Inhibition of membrane KCNK3 expression induced by hypoxia occurs mainly in VSMCs and pulmonary ECs, resulting in localized inflammation and vascular remodelling [65, 66]. Neutrophil extracellular traps are also involved in endothelial dysfunction and vascular homeostasis, which is attributed to proinflammation, pro-thrombosis, and the induction of NF-κB [121]. VEC ferroptosis in MCT-induced experimental PH induces HMGB1 release, leading to the upregulation of TLR4 expression in macrophages and triggering the inflammatory response via the HMGB1/TLR4/NLRP3 inflammasome signalling pathway [122]. Aryl hydrocarbon receptors in endothelial cells induce PH in rat models by upregulating inflammatory signals and increasing the accumulation of CD4 + cells [123]. The production of granulocyte-macrophage colony-stimulating factor in pulmonary artery VECs prevents chronically hypoxia-induced PH in mice and is upregulated when reducing BMPR2 expression [124]. In addition, decreased peroxiredoxin 6 in VECs induces the release of HMGB1 and activation of the TLR4/NLRP3 signalling pathway, thus promoting MCT-induced PH in rats [125]. The upregulated salusin-β in VECs and macrophages in PH rats has been reported to be a main contributor to both atherosclerosis and myocardial ischaemic disease, and promotes pulmonary VEC dysfunction via the activation of NF-κB signalling, thus inducing pulmonary inflammation and vascular remodelling [126].

Some factors expressed by activated macrophages can regulate vascular cell function. The proliferation of pulmonary VSMCs can be upregulated by VEGF and platelet-derived growth factor (PDGF), which are expressed by accumulated macrophages in pulmonary vessels [68, 99]. Moreover, pulmonary VSMC proliferation can be inhibited by prostaglandin D2 released by macrophages [78]. The proliferative phenotype of VSMC exhibits HIF-1α activation and enhanced glycolysis in normoxic conditions in experimental PH models [127]. Since the accumulation of VSMCs is very common in PH, the inhibition of smooth muscle cell proliferation and migration via regulating IL-1β, HIF or Nox4 has been reported to be a promising and effective therapeutic target for PH [37, 38, 69, 76]. In addition, the expression of MMP-1 and MMP-10 in M1-polarized macrophages increased in both PH patients and PH rat models, and the increased expression of MMP-1 and MMP-10 can promote the proliferation and promigratory phenotypes of VSMCs [96]. VSMC-derived TGF-β regulates the phenotypes of macrophages via p38-MAPK-dependent signalling, which in turn promotes VSMC proliferation [128]. Additionally, VSMC-derived multipotent vascular progenitor cells can differentiate into multiple phenotypes, such as mature VSMCs, resident macrophages, and endothelial-like cells [129]. These vascular progenitor cells might be derived from VSMCs and located at the normal pulmonary artery muscular-unmuscular border [130], or they can be derived from distinct PW1 + cells in perivascular zones [131].

Another typical characteristic of pulmonary vascular remodelling in PH is the presence of the proinflammatory fibroblast phenotype, as proposed by the outside-in hypothesis of pathological vascular remodelling [132]. When exposed to external stimuli such as hypoxia, fibroblasts produce increased levels of lactic acid, succinate, citrulline, IL-6, and other inflammation-related mediators, thus leading to microenvironmental alterations and anti-inflammatory effects to enhance proliferation and apoptosis resistance [82]. These proinflammatory phenotype fibroblasts exhibit increased HDAC I activity and express high levels of several typical products, such as classic cytokines (IL-1 and IL-6), macrophage chemoattractants (CCL2, CXCL12, and CCL5), the macrophage growth factor GM-CSF, and the adhesion protein VCAM-1, thus inducing the migration, adhesion, and activation of monocytes [6]. In addition, the MiR-124/PTBP1/PKM axis has been reported to be associated with metabolic reprogramming in proinflammatory fibroblasts [133]. Galectin-3 is also expressed in fibroblasts and serves as a pattern-recognition receptor and danger-associated molecular pattern in macrophages, leading to M2 polarization [134].

**Fig. 3:**
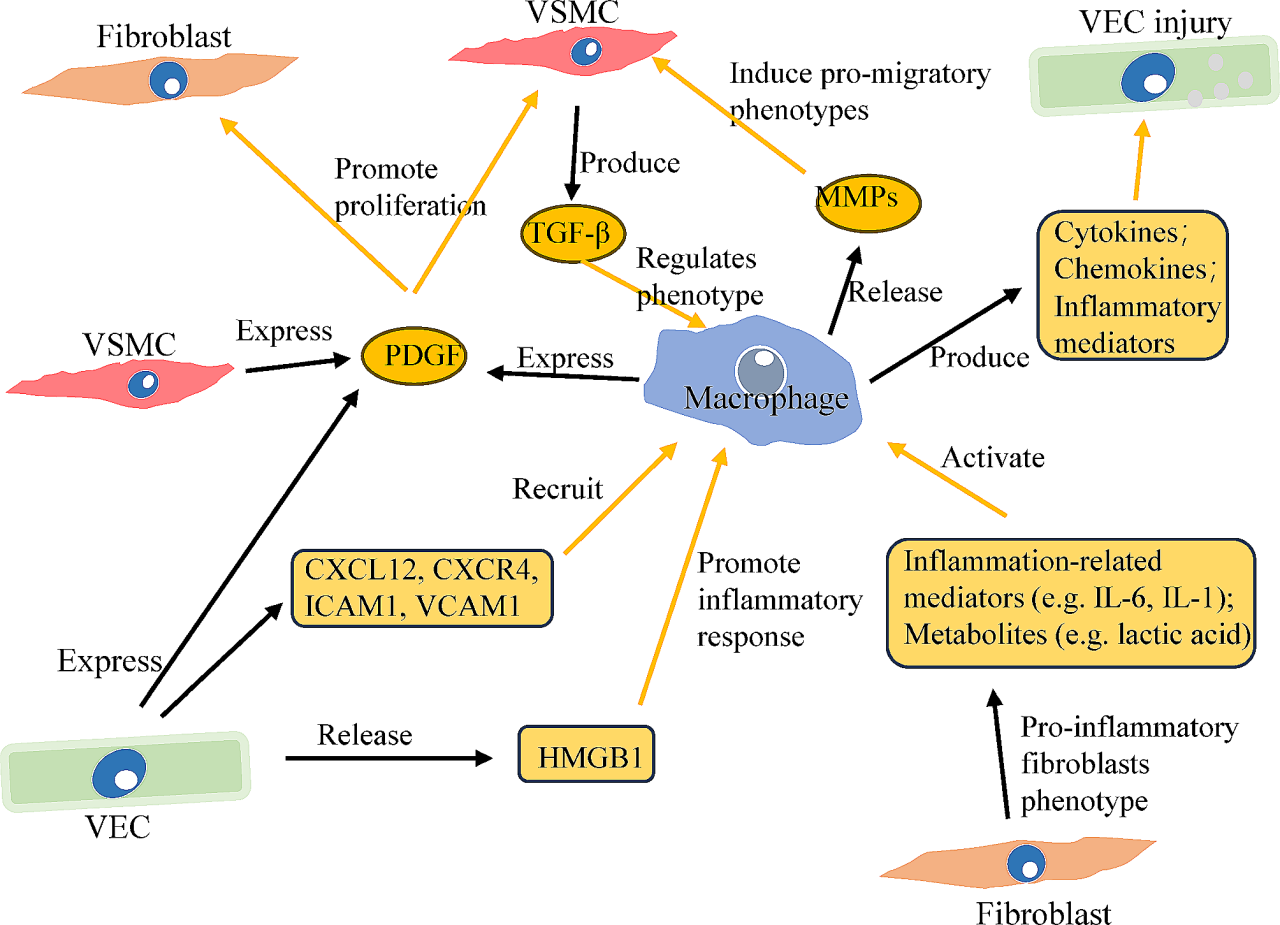
The interaction between macrophages and other vessel cells in PH pathogenesis. Although macrophages might accelerate PH progression, pulmonary artery remodelling still results from abnormal angiogenesis. VECs, VSMCs and fibroblasts induce the recruitment of macrophages upon injury, while activated macrophages release variable mediators at different stages of inflammation. Influenced by these inflammatory mediators, vessel cells alter to specific phenotypes for survival or proliferation and release specific factors, such as IL-1, TGF-β, PDGF, or chemokines. These factors can regulate macrophage function and polarization in turn. For example, TGF-β contributes to M2-like polarization, while HMGB1 contributes to M1-like polarization. Together, these complex interactions eventually result in vascular remodelling and pulmonary hypertension

Notably, fibroblasts can activate the inflammatory phenotype and immune response of BMDMs, and increase aerobic glycolysis in BMDMs [116]. In addition to cytokines and chemokines, fibroblasts can also release extracellular vesicles [135], which can provide ROS [37]. The altered microenvironment forces macrophages to react. On the one hand, increased lactic acid, succinate, and IL-6 levels drive STAT3 and HIF-1 activation, thus promoting metabolic reprogramming in macrophages, while increased citrulline serves as a material for arginase in macrophages and maintains its function [82]. On the other hand, extracellular vesicles induce the transformation of phenotypes and promote the release of mediators in BMDMs, such as IL-4, G-CSF, CXCL13, MCP-5, CXCL11, IL-16, IL-17, IL-1β, CCL5, CCL1, MIG, IL-10, IL-23, TNF-α, IL-27, IFN-γ, IL-13, MIP-2, sICAM-1, IL-3, TIMP-1, and CXCL-1 [135]. Metabolic coordination between macrophages and fibroblasts is subsequently established, leading to persistent metabolic alterations and cell activation. In addition to VECs, VSMCs and fibroblasts, which have received the most attention, other cellular components, such as functional T cells, are also reported to play a protective role in vascular remodelling in a macrophage-related manner [136]. The main interactions between macrophages and other vessel cells are presented in Fig. 3.

### Macrophages as promising therapeutic targets for PH

Current medical interventions, which involve a combination of multiple agents, focus on improving the symptoms and prognosis of PH patients [137]. PDE5 inhibitors such as sildenafil, tadalafil, and vardenafil are effective for Group 1 PH and can be delivered by oral or inhaled treatment [138, 139]. In addition, prostacyclin, endothelin receptor antagonists, and guanylate cyclase stimulators are also effective for specific PH groups [140–142]. These classical pulmonary hypertension agents may also affect macrophages. Classic PDE5 inhibitors have been reported to inhibit the mobilization and recruitment of bone marrow-derived cells as well as the release of proinflammatory cytokines [143–145]. In addition, sGC stimulators (such as Riociguat) modulate liver inflammation via the inhibition of NLRP3 inflammasome-mediated IL-1β production in Kupffer cells [146]. Endothelin receptor antagonists, such as bosentan, macitentan and ambrisentan, suppress cytokines released from AMs and thus present anti-inflammatory potential [147]. Endothelin has been demonstrated to trigger M2 macrophage accumulation and ROS formation [148, 149]. Prostanoids (including epoprostenol, treprostinil, iloprost, beraprost, and selexipag) have been recognized as major regulators of inflammation progression and resolution [150]. A recent study revealed a novel cyclooxygenase/prostaglandin E2 axis-dependent mechanism of HIF-1α-induced TNF-α expression in macrophages [151]. These studies suggest that classic pharmacological agents for PH treatment might influence the immune system to some degree.

In addition to classical PH drugs, new therapeutic targets are emerging. The protein Regnase-1 is encoded by the ZC3H12A gene and is involved in mRNA degradation [152], which suppresses PH progression by degrading the mRNA of IL-6 and PDGF in AMs [41]. The intratracheal administration of Regnase-1-targeting morpholino oligonucleotides, which enhances Regnase-1 expression, has shown therapeutic efficacy by attenuating inflammatory cascades and fibrosis [153]. However, there is a lack of available pharmacological molecules to enhance Regnase-1 expression in vivo. Serum glucocorticoid-regulated kinase 1 (SGK1), a member of the serine/threonine kinase family, is associated with macrophage activation and inflammatory response. Deletion of SGK1 inhibits macrophage infiltration in the lungs of experimental PH models [39]. EMD638683, an SGK1 inhibitor, has been reported to suppress macrophage infiltration and prevent PH progression in MCT-induced rat models [154]. LDL receptor-related protein 1 (LRP1) plays an anti-inflammatory role in several diseases by maintaining cholesterol homeostasis, inhibiting migration, and blocking cytokine release [12]. In experimental PH, the increased expression of LRP1 in macrophages attenuates neointima formation by inducing the degradation of TGF-β2 [155]. LRP1 has also been identified as an integrator of TGF-β1-mediated vascular remodelling in PH. The expression of LRP1 is important to vascular homeostasis. PH induced by LRP1 deficiency can be reversed by pharmacologic PPARγ activation with pioglitazone [156]. Other LDL receptor family members are also potential therapeutic targets for PH [156].

Since HDACs are involved in perivascular fibroblast proliferation and monocyte activation, the inhibition of HDACs is believed to be an effective therapy for PH patients. However, the efficacy of HDACs pharmacological inhibitors in PH remains a controversial issue [6, 133, 157, 158]. Sotatercept, a ligand trap for multiple TGF-β family members, can suppress macrophage infiltration and reverse experimental PH [159]. The neutralization of CXCL12 improved pulmonary hemodynamics and structural disorders in both the lungs and heart in rat PH models [117]. Donepezil is an acetylcholinesterase inhibitor with a parasympathetic activistic effect. By suppressing M2-macrophage activation, donepezil reverses VSMC dysfunction in MCT-induced rat PH models [160]. The antifibrotic agent pirfenidone decreases macrophage IL-1β secretion in vitro [161].

Similar metabolic remodelling occurs in both fibroblasts and macrophages during PH pathogenesis, which indicates that short- or long-term metabolic regulation may potentially affect PH progression [82]. MTOB, an inhibitor of CtBP1, has been reported to attenuate glycolysis and inflammatory gene expression in macrophages and fibroblasts, thus leading to reversion to hypoxia-induced vascular remodelling and perivascular macrophage accumulation [116, 162]. Carbonic anhydrase inhibitors could also modulate AM activation and polarization and restore vascular homeostasis [163]. Molecular hydrogen restored the increased expression of MCP-1 and stromal cell-derived factor-1 in the MCT-induced PH model and thus suppressed adventitial macrophage accumulation [164]. A new series of N-(phenylmethyl)-benzoxazole-2-thiones, which act as MIF antagonists, have been reported to successfully reverse established MCT-induced PH and alleviate hemodynamics [165]. Additionally, Cheng et al. reported that a novel synthesized hybrid can both reduce the proliferation of perivascular cells and alleviate macrophage infiltration, thus attenuating MCT-induced PH in rat models [166]. Several researchers have attempted to prevent PH by injecting tolerogenic macrophages generated from monocytes into athymic nude rats [167]. Dynamin-related protein 1 induces both a polarity shift and inflammatory mediator expression in macrophages after vascular injury, resulting in intimal thickening [168], which is similar to vascular remodelling in PH. These studies suggest that regulating mitochondrial function in macrophages might be a promising therapeutic target for vascular diseases. In addition, several novel methods of drug delivery have been tested, offering the possibility of tissue-selective delivery [169, 170].

Numerous agents are being tested in clinical trials. These novel agents are summarized in Table 4. Although not all agents are specifically designed to target monocytes or macrophages, they might still affect macrophage function. Targeting mTOR signalling in macrophages has been demonstrated to be a potential therapeutic intervention in cardiovascular diseases [171]. AMP-activated protein kinase activation in macrophages suppresses the inflammatory response [172]. Recombinant relaxin peptides delivered by inhaled porous microspheres can suppress macrophage M2 polarization [173]. Dapagliflozin has been previously tested for its ability to attenuate inflammation and regulate macrophage polarization in cardiac fibrosis [174]. Besides the classical TGF-β and SMAD signalling pathways, activin and PDGF receptor-β are associated with macrophage activation and recruitment [175, 176]. Lysyl-tRNA synthetase induces immune responses through the activation of monocytes and macrophages [177]. Recently, a lysyl-tRNA synthetase (KARS1) inhibitor, ZMA001, is being evaluated as a potential PH therapy. The KARS1 inhibitor ZMA001 is designed to block KARS1-dependent infiltration of monocytes/macrophages and inhibit inflammatory responses in vessels (NCT05967299, registration date: August 1, 2023).

**Table 4:** Novel PH agents are being tested

Agent	Function	Phase	Classification	Study ID and registration date
LAM-001	mTOR inhibitor	Phase 2	Group-1 or -3	NCT05798923 (April 5, 2023)
Metformin	AMPK activator	Phase 2	Group 2	NCT03629340 (August 14, 2018)
AZD3427	relaxin mimetic agonist	Phase 2	Group 2	NCT05737940 (February 21, 2023)
MK-5475	sGC stimulator	Phase 2	Group-3	NCT05612035 (November 10, 2022)
Dapagliflozin	SGLT2 inhibitor	Phase 2	Group-1 or -4	NCT05179356 (January 5, 2022)
LTP001	SMURF1 antagonists	Phase 2	Group-1	NCT05135000 (November 26, 2021)
Sotatercept	activin signalling inhibitor	Phase 2, 3	Group-1 or -2	NCT04945460 (June 30, 2021), NCT04896008 (May 21, 2021), NCT04811092 (March 23, 2021), NCT05587712 (October 20, 2022), NCT04796337 (March 12, 2021)
KER-012	TGF-β inhibitor	Phase 2	Group-1	NCT05975905 (August 4, 2023)
iMatinib	tyrosine kinase inhibitor	Phase 3	Group-1	NCT05557942 (September 28, 2022)
Seralutinib	PDGFRα and PDGFRβ inhibitor	Phase 2, 3	Group-1	NCT04816604 (March 25, 2021), NCT05934526 (July 7, 2023)
Ralinepag	prostacyclin receptor agonist	Phase 3	Group-1	NCT03683186 (September 25, 2018), NCT03626688 (August 13, 2018)
ZMA001	Lysyl-tRNA synthetase inhibitor	Phase 1	Healthy volunteers	NCT05967299 (August 1, 2023)

Abbreviations: mTOR, mammalian target of rapamycin; AMPK, AMP-activated protein kinase; sGC, soluble guanylate cyclase; SGLT2, sodium/glucose cotransporter 2; SMURF1, SMAD specific E3 ubiquitin protein ligase 1; TGF-β, transforming growth factor-β; PDGFR, platelet-derived growth factor receptor

## Conclusion

Recent advances have revealed the role of macrophages as key regulators of PH pathogenesis. When exposed to PH triggers, macrophages are recruited and then differentiate into different phenotypes at specific time points, inducing perivascular inflammation, endothelial dysfunction, and consequent vascular remodelling. Macrophages are involved in many typical hallmarks of PH, such as smooth muscle cell proliferation and fibroblast activation, which are essential for PH development. Inflammation-related soluble mediators are closely linked to these alterations. M1 and M2 macrophages are commonly treated as signs of different stages in PH pathogenesis. M1 macrophages promote inflammation, while M2 macrophages have anti-inflammatory functions and regulate tissue repair. Interestingly, M2 macrophages are regarded as promotors of advanced PH in most studies due to their wound-healing function, and M1 macrophages play protective roles in PH in specific circumstances [178]. Several novel macrophage targets have been reported in preclinical studies. However, these novel therapeutic targets need to be tested in further investigation. Macrophages are now recognized as candidate therapeutic targets for PH treatment due to their unique role in PH pathogenesis, suggesting a new strategy for preventing and even reversing PH progression.

## Data Availability

Not applicable.

## Abbreviations

AMs: Alveolar macrophages
BMDMs: Bone marrow-derived macrophages
BMPR2: Bone morphogenetic protein receptor 2
BTK: Bruton’s tyrosine kinase
CtBP1: C-terminal binding protein 1
DAMP: Damage-associated molecular pattern
EIF2: Eukaryotic translation initiation factor 2
EGFR: Epidermal growth factor receptor
FIZZ1: Found in inflammatory zone
G6PD: Glucose-6-phosphate dehydrogenase
GM-CSF: Granulocyte-macrophage colony-stimulating factor
HDAC: Histone deacetylase
HERV-K: Human endogenous retrovirus K
HIF: Hypoxia-inducible factor
HIMF: Hypoxia-induced mitogenic factor
HMGB1: High mobility group box-1 protein
Hmox1: Heme oxygenase 1
IMs: Interstitial macrophages
IRS2: Insulin receptor substrate 2
KARS1: Lysyl-tRNA synthetase
KCNK3: Potassium channel subfamily K member 3
LRP1: LDL receptor-related protein 1
LTB4: Leukotriene B4
MAPK: Mitogen-activated protein kinase
MCP: Monocyte chemoattractant protein
MCT: Monocrotaline
MIF: Migration inhibitory factor
mPAP: Mean pulmonary arterial pressure
mTOR: Mammalian target of rapamycin
mTORC1: mTOR complex 1
NF-κB: Nuclear factor kappa-B
NLRP3: NOD-like receptor thermal protein domain associated protein 3
Nox4: NADPH oxidase 4
P2X7R: P2 × 7 purinergic receptor
PDE5: Phosphodiesterase 5
PDGF: Platelet-derived growth factor
PH: Pulmonary hypertension
Pfkfb3: 6-phosphofructo-2-kinase/fructose-2,6-bisphosphatase 3
PKM: Pyruvate kinase muscle
PLC: Phospholipase C
PTBP1: Polypyrimidine tract binding protein 1
RAGE: Receptor for advanced glycation end products
RELM-α: Resistin-like molecule-α
ROS: Reactive oxygen species
RUNX1: Runt-related transcription factor 1
sGC: Soluble guanylate cyclase
sICAM-1: Soluble intercellular adhesion molecule-1
SIRT1: Sirtuin 1
SGK1: Serum glucocorticoid-regulated kinase-1
STAT-3: Signal transducer and activator of transcription 3
TIMP-1: Tissue inhibitor of metalloproteinase 1
TGF-β: Transforming growth factor-β
TLR4: Toll-like receptor 4
VCAM-1: Vascular cell adhesion molecule-1
VECs: Vascular endothelial cells
VEGF: Vascular endothelial growth factor
VSMCs: Vascular smooth muscle cells
Ythdf2: YT521-B homology domain family 2
